# Mr. Jones, of Jersey

**Published:** 1861-11

**Authors:** 


### NECROLOGICAL NOTICES.

MR. JONES, OF JERSEY.—We deeply regret to have to announce the death of this surgeon, which took place on Saturday last, from the effects of typhus fever, caught in the discharge of his duties. Some three or four years ago, Mr. Jones was seized with partial paralysis and great prostration of the system, after having sat up for nearly thirty nights in succession, in attendance upon an old friend and patient. From the effects of this attack he never thoroughly recovered, although he benefited very much by a total abstinence from professional duties for some time, and by the care and attention bestowed upon him by his friend, the late Dr. Todd. He, however, returned to his practice, and entered upon his duties with undiminished ardor, but with a frame not capable of resisting contagion as it did formerly, and he died at his post, working to the last, as it became the man, and probably as he would have wished.

Perhaps there has been no surgeon out of the three metropolitan cities who has attracted greater attention, during the last ten years, than Mr. Jones. Up to 1850, his name was not known beyond the little island which was the scene of his surgical exploits; but about that time he signalized himself by the performance of several remarkably successful cases of excision of joints, especially of the knee, and by his practice and writings he contributed as much as any one to the rapid spread of conservative surgery. So anxious was Mr. Jones to encourage the operation of excision of the knee-joint instead of amputation, that he was at the trouble and expense of coming over to England with some of the poor patients upon whom he had operated, and a great impression was thus made in favor of this proceeding, about the merits of which there was at this particular time a great deal of warm discussion. In connection with this subject, it is right to mention that, although most undoubtedly the merit of reviving excision of the knee is due to Mr. Fergusson, the subject of this memoir adopted the operation a few weeks after Mr. Fergusson had first performed it, but without being aware of the fact. The success attending the operations of Mr. Jones was remarkable. When the writer of this notice last visited Jersey, Mr. Jones had performed fifteen excisions of the knee, with only one death. He performed on numerous occasions the excisions of the other joints with almost equal success, especially those of the hip and ankle. The most perfect result from the latter operation we have ever seen, was in the person of an adult operated upon by Mr. Jones a few years ago.

The well-known success following Mr. Jones's operations has been often attributed to the pure air of Jersey; but those who, like the writer, have had opportunities of watching Mr. Jones's practice, would be able to affirm with truth that the results were more owing to the assiduous care bestowed by the operator in the after-treatment of his cases. The hospital where most of his cases were operated upon was not well calculated for such a purpose; the wards were small, the beds were close together, and the ventilation was by no means good. Mr. Jones, however, used to support his patients well after operations, watched symptoms narrowly, and was most careful and gentle in dressing wounds. Hence the reason for the extraordinary success which undoubtedly followed his great surgical operations, and for the reputation which he most deservedly acquired during the last ten years of his life. His practice was considered by some to be bold, even to the verge of rashness, as, for instance, in the exhibition of half-ounce doses of tincture of digitalis for delirium tremens; but the results justified the practice.

His loss will be felt severely in the Island of Jersey, both by poor and rich, among whom he practiced for a period of thirty-five years; and those who knew him as a friend will readily admit that it will be difficult to find a man with a more thoroughly genial, honest, and unselfish nature.

The *Jersey Times* says of Mr. Jones, that his memory will be cherished as the memory of a kind and humane man—"A man who always had an encouraging word and a kindly smile for the poorest patient under his care—always a gentleness and a fatherly tenderness for the sick children sprinkled about the wards of the hospital.

"To record the fact of Mr. Jones's death is a work almost of supererogation; for the sorrowful tidings having spread with a rapidity which marks the universal reputation he had earned, as truly as the unqualified expression of regret which attended the information marks the deep respect in which he was held."—*London Med. Times and Gazette*, Sept. 14, 1861.

